# An early gall-inducing parasitic wasp adversely affects the fitness of its host *Ficus* tree but not the pollinator

**DOI:** 10.1038/s41598-020-71738-9

**Published:** 2020-09-10

**Authors:** Xiao-Wei Zhang, Liang-Heng Li

**Affiliations:** 1 College of Life Sciences, Shaanxi Normal University, Xi’an, 710119 Shaanxi People’s Republic of China; 2 Center for Ecological and Environmental Sciences, Northwestern Polytechnical University, Xi’an, 710072 Shaanxi People’s Republic of China

**Keywords:** Evolutionary ecology, Population dynamics, Tropical ecology, Ecology

## Abstract

The fig tree-fig pollinator mutualism is one of the most tightly knit symbiotic systems. The research on the ecology of non-pollinators which exploit the mutualism without providing services to the host is very limited and conclusions about the role they play in the maintenance of this mutualism are full of contradictions. The non-pollinating fig wasps species are highly diverse in their feeding habit and ecological function, which may result in different consequences on the mutualism. *Sycophaga testacea* is an early-ovipositing galler hosted by *Ficus racemosa*, which is a potencial competitor to the pollinators as they use the same female flowers in the fig as their ovipositing sites. In this study, we investigate the effect of *S. testacea* on the production of both pollinator and fig tree with a field control experiment. Seed production is decreased significantly when the figs were parasitized, while the offspring production of the pollinator is not significantly affected, which indicates that this galler species has a harmful effect on the fitness of its host fig tree but not the pollinator. The overall development ratio of the galls is decreased significantly when the figs were parasitized, and we found that the intrinsic low development ratio of *S. testacea* is responsible for the decrease in the overall development ratio.

## Introduction

Parasites exist in almost all mutualisms. They exploit resources involved in the mutualism without providing a benefit. It is a central problem for evolutionary biology to address what role the parasites play in the maintenance of cooperation of a mutualism^1–3^. Parasites have an obvious destructive effect on the mutualism, and explaining how parasites are kept from driving mutualisms extinct remains a challenging unsolved problem^1^. Among the various kinds of mutualisms, the obligate mutualism of fig trees (*Ficus spp*) and their pollinating fig wasp is a most extensive studied one^2,4,5^. The fig or syconium is the highly specialized enclosed inflorescence of *Ficus*. The ostiole is a tunnel formed by overlapping scales and it’s the only way to enter a syconium^4^. Usually only the pollinators can enter and pollinate the flowers inside the syconia^5^. The pollinating fig wasps complete their life cycle within the syconia by utilizing the nutrition of the female flowers inside the syconia, and for fig trees, they are usually the only agent capable of pollinating syconia^4^. A flower that receives an egg deposited by the pollinating fig wasp forms a gall and develops a wasp offspring, and flowers that do not receive a wasp egg and are pollinated develop into seeds.

Except for the pollinators, multiple non-pollinating fig wasp (NPFW) species are also hosted by fig-trees^2^. The syconia of most *Ficus* spp generally host a single pollinator species and several fig species-specific parasitic, non-pollinating fig wasps^2,6,7^, which oviposit at different stages of syconia development according to their different feeding habit^8,9^. Most of the NPFW species lay their eggs from outside of the fig into the flower with their long ovipositor. The NPFWs range from 1 to 36 species hosted by a single *Ficus* species^10^ and can be divided into several categories according to their special feeding habit, which are gallers, parasitoids, and inquilines^1^. Gallers produce galls by themselves when they oviposit in fig flowers, and their larvae feed on tissue of galled ovaries^11^. Parasitoid larvae feed on the larvae of pollinators or gallers, and inquilines feed on the gall tissue induced by either galler or pollinator larvae.

Diverse species of non-pollinators co-exist within a fig inflorescence, and the knowledge on biology and ecology of the non-pollinating wasp community is very limited for us^12^. NPFW species may have different effects on the reproduction of fig tree and pollinator. Some studies found that NPFWs can reduce both seed production and the number of pollinating wasps^9,13,14^, other studies found that NPFWs only reduce the number of pollinating wasps^11,15–18^, and some studies found that NPFWs have no negative effect on the pollinating wasps^19,20^. For example, by collecting and analyzing the natural maturing fruits that contain both gall inducer species *Anidarnes bicolor* and pollinators, Bronstein^15^ found that the number of pollinator offspring decreased significantly with the increase of the number of *A. bicolor*, but no reduction in seed production occured. Conchou et al.^13^ analyzed the effect of large-sized NPFWs on the production of pollinators and seeds through a controlled experiment. In their study, they clarified the community structure and feeding habits of NPFWs hosted by *Ficus guianensis*, and found a significant negative correlation between the number of NPFW and the production of pollinators and seeds^13^. The former studies have shown that the gall inducer can have a substantial impact on the reproductive success of their host fig tree and the pollinators. The community structure for *F. racemosa* in South China and India is known clearly, in which two (three in India) gall inducers oviposit sequentially, following by two parasitoids of the gall inducers and a parasitoid of the pollinator^21–23^. Although the non-pollinator community structure of *F. racemosa* is well studied, the effect of each species on the fitness of *F. racemosa* and its pollinator remains unknown.

The NPFWs of different feeding habits may have different effects on the mutualism. In order to get a better understanding of the effects of NPFWs, we need to do more controlled accurate experiments. In this research, we studied the effect of early gall-inducing non-pollinator on the fate of this mutualism using an early-ovipositing galler species *S. testacea* and pollinator species *Ceratosolen fusciceps* that hosted by *Ficus racemosa*.

## Results

### Size of mature figs

**Figure 1:**
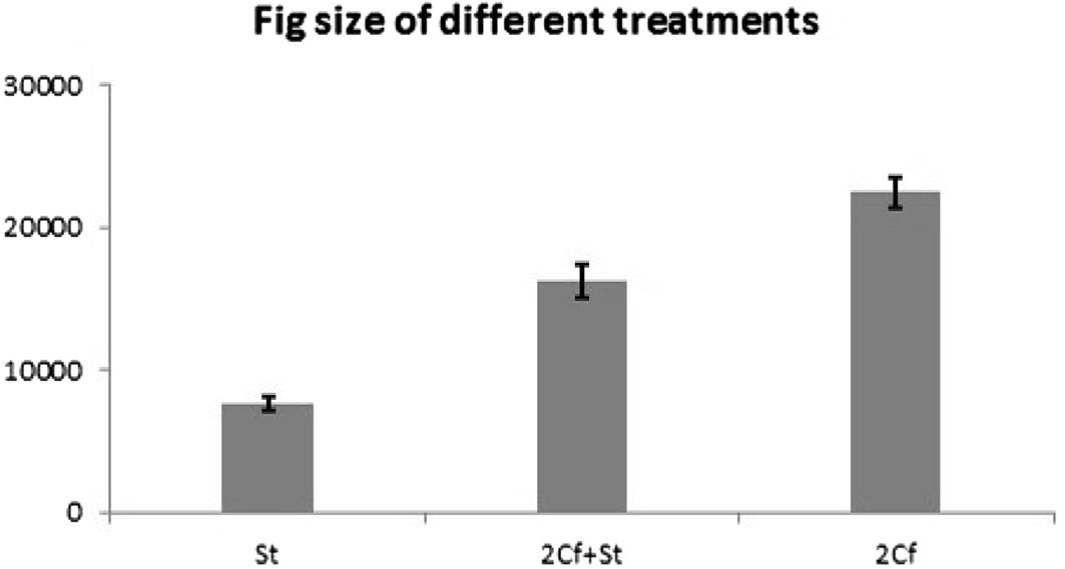
The fig size increases significantly with the increase in the proportion of pollinator. (One-way ANOVA of fig volume among three treatments: F_2,46_ = 80.213, *P* < 0.001): St treatment: Mean ± SE = 7,574.06 ± 505.31, N = 20; 2Cf + St treatment: Mean ± SE = 16,139.03 ± 1,161.11, N = 10; 2Cf treatment: Mean ± SE = 22,435.3 ± 1,085.09, N = 19.

The experiments have three different treatements. To know and compare the growth of the figs in different treatments, the size of mature figs were measured. We added the length and the 2 diameters (mm), then divided the sum by 6 to represent the radius (r) of the fig. The volume of the fig was calculated as a sphere (Volume = 4/3*π*r^3^). The size of mature figs varied significantly among treatment (F_2,46_ = 80.213, *P* < 0.001; Fig. 1).

The size of mature figs in which only non-pollinating fig wasp *S. testacea* had oviposited was much smaller than that of figs into which pollinators had been introduced. The figs into which both *S. testacea* and *Ceratosolen* had oviposited were intermediate in volume and those into which only the pollinators had been introduced were largest.

### Development ratio

**Figure 2:**
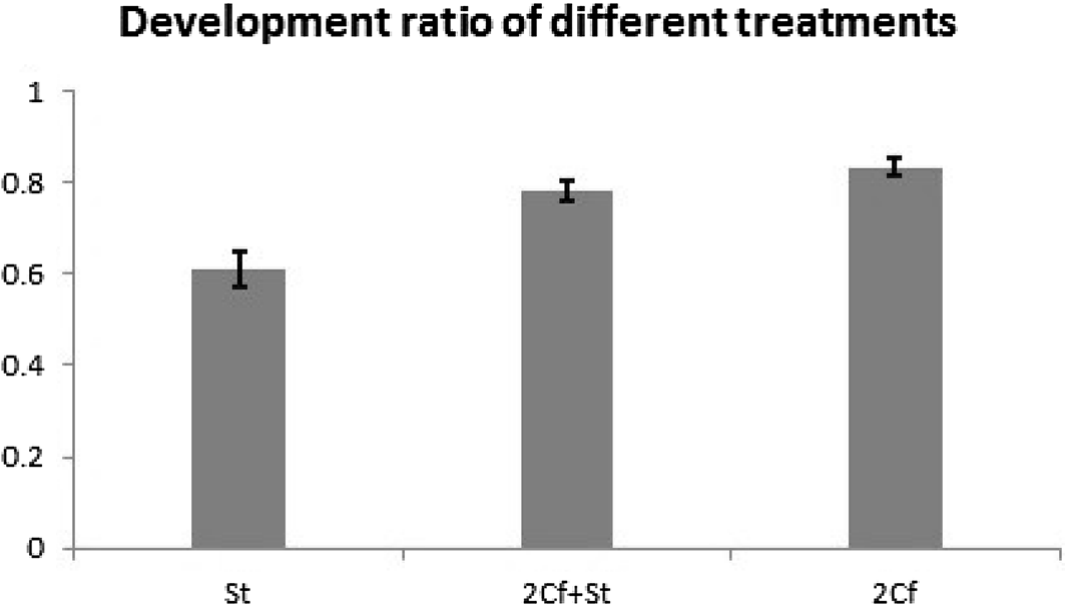
The development ratio improves significantly with the involvement of pollinator. (One-way ANOVA of development ratio among three treatments: F_2,46_ = 15.189, *P* < 0.001): St treatment: Mean ± SE = 0.61 ± 0.04, N = 20; 2Cf + St treatment: Mean ± SE = 0.78 ± 0.02, N = 10; 2Cf treatment: Mean ± SE = 0.83 ± 0.02, N = 19.

Development ratio reflects the percentage of the galls that produce wasps successfully. The development ratio (the number of wasps divided by the number of galls) shows the same relationship as fig volume (F_2,46_ = 15.189, *P* < 0.001; Fig. 2). The development ratio was lowest when only non-pollinating fig wasp *S. testacea* had oviposited in the fig, was intermediate when both pollinators and *S. testacea* had oviposited in the figs and was highest when only pollinators had oviposited in the fig.

### Numbers of galls and wasp offsprings

**Figure 3:**
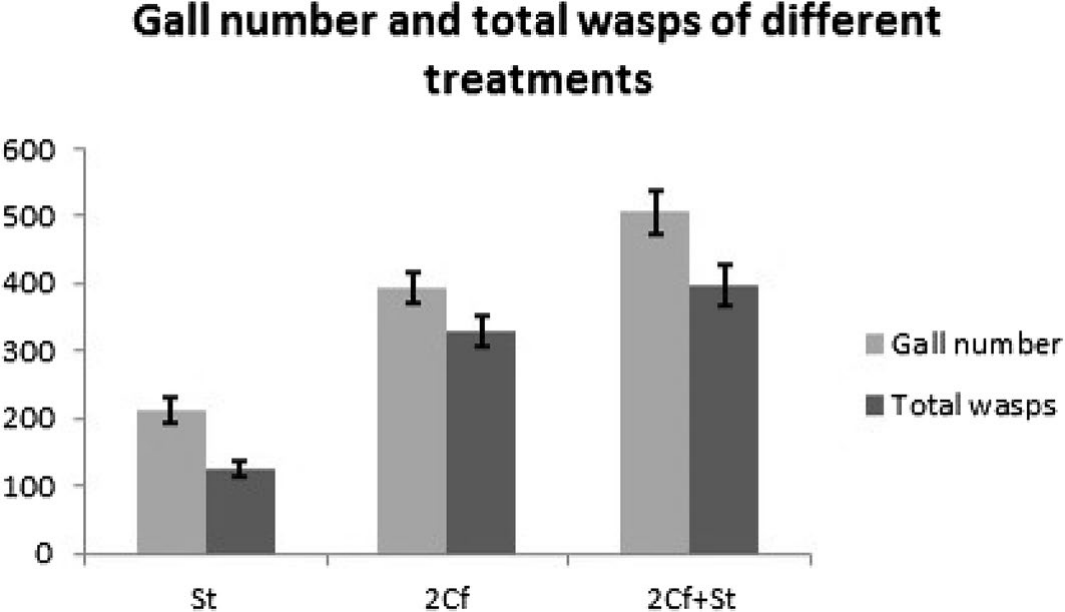
The gall number and total wasps show the same trends in three different treatments. (independent T-test of gall number between 2 and 2Cf + St treatment: t = 2.824, df = 27, *P* < 0.05): 2Cf treatment: Mean ± SE = 393.68 ± 23.02, N = 19; 2Cf + St treatment: Mean ± SE = 505.70 ± 32.823, N = 10; (independent T-test of total wasp number between 2 and 2Cf + St treatment : t = 1.72, df = 27, P = 0.097): 2Cf treatment: Mean ± SE = 331.00 ± 22.673, N = 19; 2Cf + St treatment: Mean ± SE = 396.40 ± 29.782, N = 10.

For figs in which only *S. testacea* had oviposited, the total number of galls was much lower than in figs into which pollinators were introduced. For the treatments with pollinators, figs with *S. testacea* contained a significantly higher number of galls than figs without *S. Testacea* (t = 2.824, df = 27, *P* < 0.05; Fig. 3).

The wasp production shows the same trends with the total number of galls. For figs in which only *S. testacea* had oviposited, the total number of waps produced was much lower than in figs into which pollinators were introduced. For the treatments with pollinators, figs with *S. testacea* contained slightly more total wasps than figs without *S. testacea*, but the difference is not significant (t = 1.72, df = 27, *P* = 0.097; Fig. 3).

### Number of pollinator offspring and seed production

**Figure 4:**
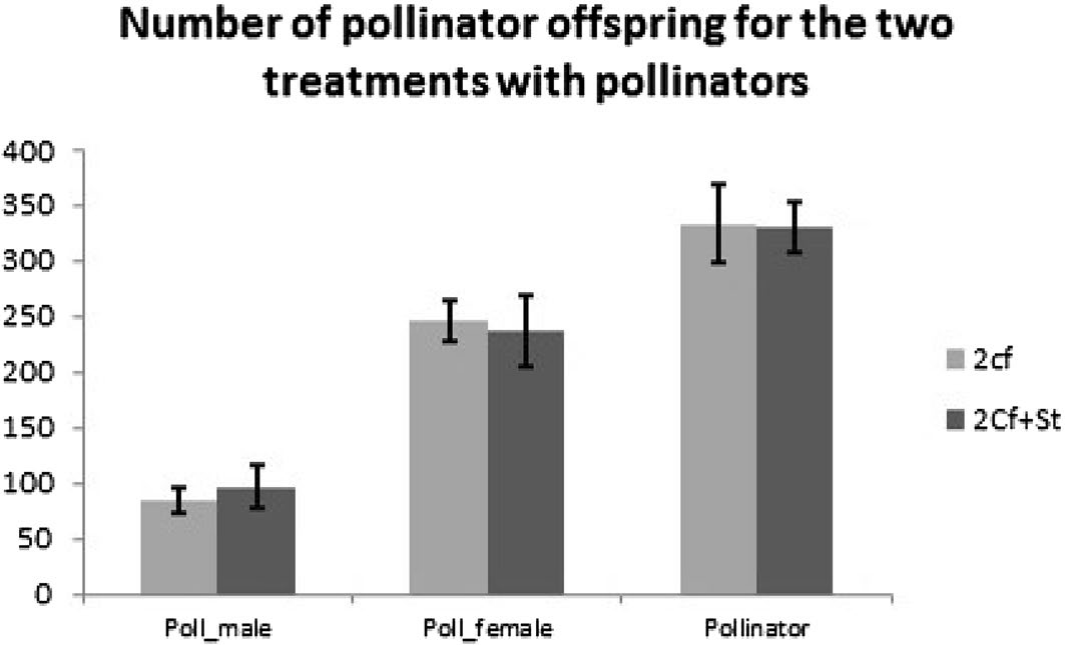
The pollinator offspring production is similar between the two treatments with pollinator foundress. (independent T-test of pollinator offspring number between 2 and 2Cf + St treatment: t = 0.063, df = 27, P = 0.951): 2Cf treatment: Mean ± SE = 333.5 ± 34.496, N = 19; 2Cf + St treatment: Mean ± SE = 331.00 ± 22.673, N = 10.

The number of pollinator offspring and seed production reflects the fitness of pollinator and fig trees respectively. The number of pollinator offspring did not differ significantly between treatment showing the limited effect of *S. testacea* on pollinator production (t = 0.063, df = 27, *P* = 0.951; Fig. 4).

The number of seeds produced by two pollinating wasps was strongly reduced in fig parasitized by the *S. testacea* (1970.95 ± 149.478 versus 1,152.6 ± 190.163 (Mean ± SE), t = − 3.297, df = 27, *P* < 0.01).

### Predicted gall number of 2Cf + St treatment

To test whether the development of pollinator and *S. testacea* dependent to each other or not, we calculated the theoretical gall number of 2Cf + St treatment and compared it with the actual value. We computed a predicted gall number of 2Cf + St treatment based on the number of *Sycophaga* divided by its averaged development ratio (0.61) in St treatment plus the number of Pollinator divided by its averaged development ratio (0.83) in 2Cf treatment (Predicted gall number = *Sycophaga* number/0.61 + Pollinator number/0.83, both *Sycophaga* number and pollinator number are from 2Cf + St treatment). The predicted gall number for 2Cf + St treatment is very similar to the actual gall number (504.92 ± 36.10 vs 505.7 ± 32.82 (Mean ± SE), t = − 0.016, df = 18, *P* = 0.987).

## Discussion

NPFWs are generally considered to have negative effects on the fig tree–fig pollinator mutualism according to theoretical studies^2,4,6^. Many empirical and experimental studies have justified this conclusion. Some studies show that NPFWs have a negative effect and found a negative correlation between the offspring number of NPFWs and the offspring number of pollinator and seeds^9,13,14^. For instance, Patel^19^ found no correlation between the offspring number of pollinator and NPFWs in their survey , and Peng et al.^20^ recorded a positive correlation between the offspring number of NPFWs and pollinator in figs of *F. hispida* . Our capacity to detect the real cost of development of different types of NPFWs and their exact effect on the mutualism is limited by many interaction factors^13^ and non-experimental empirical studies can underestimate or fail to detect the impact of NPFWs on this obligate mutualism when overall consideration was taken with different studies of pollinator-NPFW interactions in consideration^18^.

For early-ovipositing gallers, a remarkable feature of those species is that their galls usually fill the space of B-phase syconium cavities and can greatly hinder pollinator movement and oviposition, thus result in their specific consequences on the mutualism^24^. For example, Conchou et al.^13^ studied *Ficicola spp.* which is a genus of early-ovipositing large-sized gallers hosted by *F. guianensis* and found a substantial negative impact of this genus on the production of both pollinators and seeds. Here, we present another case that studies the effect of an early gall inducer.

Our study has explored the effect of early-ovipositing gallers on the growth of figs (fig size of matured figs) for the first time. In our study, it turns out that early-ovipositing gallers can greatly depress the growth of the figs. The mechanical injury caused by the insertion of the ovipositor of *S. testacea* to the tiny figs may be responsible for the depression of the growth of the Fig^25^. Another possible reason is the gall-inducing process^26^. Jansen-González^26^ et al. studied the gall-inducing process and larval feeding strategy of pollinating and non-pollinating fig wasp species associated with *F. citrifolia* and found that the way non-pollinating galler induces gall is quite different from pollinators and they exploit plant resources more aggressively^26^. With the involvement of the pollinators, the growth of figs improved greatly. The existence of both seeds and larvae of the pollinator, both of which is essential to the fitness of fig trees, may contribute to the improvement of the growth of the figs.

The variation trend of development ratio is consistent with fig volume, which can be explained by the saction mechanism applying to uncooperative cheaters^27–31^. Jander et al.^27–29^ found that the *Ficus* tree can sanction the pollen-free pollinators through decreasing their offspring development ratio and increasing the abortion rate of the unpollinated figs. Almost all the non-pollinating figs wasp species lay their eggs in the female flowers but not spread pollen to the fig, which is analogous to the cheaters of pollinators. It makes sense that the same sanction mechanism works on the non-pollinating fig wasps. When only *S. testacea* oviposit in the fig, most of the figs aborted, and the fig tree invests little nutrition for the remaining figs that survived. With the increase of oviposition and pollination by pollinators (2Cf + St treatments versus St treatment), and the decrease of mechanical injury by NPFWs (2Cf treatments versus St treatment), fig trees invest more nutrition to those figs and the development ratio of the galls increases. Jander et al.^28^ argued that sanctions can be modular or individual. In a modular sanction, all the offspring produced in the fruit is punished due to involvement of non-cooperative individuals. Our experiment can be a good test of this hypothesis as we can investigate accurately the development of the pollinator and the cheater (*S. testacea*) respectively. By comparing the theoretical value and the actual number of galls for 2Cf + St treatment, we can explore the source that leads to the decrease of the overall development ratio in the 2Cf + St treatment compared with the 2Cf treatments, thus determine if the sanction works at fig level (modular) or not. We found there is no significant difference between theoretical value and the actual number for 2Cf + St treatment, which suggests that the development ratio of the two species is independent of each other, so the lower development ratio of 2Cf + St treatment compared with 2Cf treatment was caused mainly by the low development ratio of *S. testacea.* For 2Cf + St treatment, the development ratio of pollinator didn’t decrease, and being pollinated didn’t change the development ratio of *S. testacea*, which means pollination didn’t increase the nutrition provided to galls of. *S. testacea.* Our results show that the sanction is not at a fig level, which is inconsistent with the sanction to the cheaters in pollinators (pollen-free pollinators)^28,30^. Perhaps the sanction mechanism is related to the identification of the cheaters. When the host can identify the cheaters, the sanction can work at individual level where they may only sanction the offspring of cheaters, such as *S. testacea* in this study. If the host can’t identify the cheaters, the sanction may work at fig level (modularly) through sanctioning all the wasps reproduced in the fig, such as the cheaters in the pollinators.

The production of seeds is influenced greatly by the wasp *S. testacea*. A former study shows that galls produced by early-ovipositing gallers often fill B-phase syconium cavities and hinder pollinator movement and oviposition^24^, which can explain the decline of the seed production. In our study, the oviposition behavior of pollinators was much less affected than the pollen spreading behavior due to the block of galls produced by *S. testacea*.

For the treatments with the pollinators, figs with *S. testacea* (2Cf + St treatment) have more galls than figs without *S. testacea* (2Cf treatment). This indicts that the existence of galls produced by *S. testacea* has little effect on the egg-laying behavior of the pollinator. Since pollinators lay their eggs in the female flowers one by one and the number of eggs to be laid is much less than the number of pollen to be spread, it’s easy to understand that oviposition is less affected than pollen spreading.

Our study shows that *S. testacea* has an obvious negative effect on the fig tree-fig pollinator mutualism. Oviposition by *S. testacea* leads to a drastic decrease in seed production, thus may harm the maintenance of stability of fig tree-fig pollinator mutualism if the fig is excessively parasitized by *S. testacea*. We think the mechanical injury from oviposition, the galling process, and the blocking of cavity by the galls of *S. testacea* may be responsible for the negative consequences. For figs in which only *S. testacea* oviposit, the total galls are much less than figs in which pollinators were introduced. There are two possible reasons. One possible reason is that the low density of the population of *S. testacea* results in the low oviposition rate. Another possible reason is that the fig tree may sanction the unpollinated figs, ie, the figs in which only *S. testacea* oviposit. Wang et al.^30,31^ found that the sanction mechanism also works on *F.* racemosa. Further more, they found that the sanction strength became stronger with an increase in foundresses^30^ (the wasps that enter the figs to oviposit). If too many *S. testacea* oviposit in figs, the figs are aborted more easily due to sanction by the tree. Only figs that contain a small number of galls survive.

Although many studies^9,11,13–18^ have shown that NPFWs can have a negative effect on the fig tree-fig pollinator mutulism due to the reduction in pollinator offspring or the seed production, some study has found that the NPFWs also can play a positive role in maintaining the stability of this obligate mutualism^32^. For example, parasites may stabilize and maintain the fig and fig wasp system through their effects on within- and between-tree reproductive phenology^32^. More specifically, oviposition by NPFWs can result in the asynchrony of the development of the figs, and increase the probabilities of pollinators finding oviposition sites, which is good for the maintenance of this mutualism^32^.

## Methods

### Material

*Ficus racemosa* is a large free-standing tree species (up to 25 m in height), which is widely distributed from the Himalayan foothills to Australia^23^. *Ficus racemosa* (subgenus *Sycomorus*) is a monoecious fig species with both seed and pollinating wasp offspring produced within the same fig. This fig species bears crops of large spherical figs (up to 45 mm in diameter when mature) on short branches (racemes) that are attached to the trunk and larger branches. The flowering pattern of *F. racemosa* is typical for monoecious *Ficus*, with intra-tree synchrony and inter-tree asynchrony^2^. It takes 40 to 50 days for the fig to develop from the appearance of fig buds to fig ripening, and each tree can bear fruits 4 to 5 times per year at Xishuangbanna Tropical Botanical Garden, Chinese Academy of Sciences (XTBG). *Ficus racemosa* is actively pollinated at XTBG by *C. fusciceps*^23^.

The figs of *F. racemosa* become receptive after about two weeks of development. When the female flowers are receptive to pollen^33^, the figs emit volatiles that attracts female *C. fusciceps* wasps which enter the fig, oviposit and pollinate. Each wasp offspring develops within a galled fig ovule, in synchrony with seed development. When the figs mature, adult wasps emerge into the fig cavity, pollinator males chew a hole through the fig wall releasing the newborn pollen loaded mated females^4,5^.

At our study site, five non-pollinating fig wasp (NPFW) species reproduce in the figs of *F. racemosa*^21^: three *Sycophaga* species (*S. mayri, S. testacea,* and *S. agraensis*) and two *Apocrypta* species (*Apocrypta sp.* and *A. westwoodi*). Those NPFW species oviposit into the ovules of female flowers from the outer surface of the fig by inserting their long ovipositors at different periods of fig growth. *Sycophaga testacea* and *S. mayri* are gall-inducers. The other three species are parasitoids. *Sycophaga agraensis* is a parasitoid of the pollinator. *Apocrypta sp.* and *A. westwoodi* are parasitoids of *S. testacea* and *S. mayri*, respectively^21^. Our study focused on the gall inducer *S. testacea. Sycophaga testacea* that oviposits the earliest among NPFW species produces large galls that usually fill the cavity of the fig during receptive stage. According to our observation, *S. agraensis* begins ovipositing when the figs just grow 3–5 days on the branch and the duration of oviposition on figs can last 2–3 days in the wet season. Strong oviposition by *S. testacea* results in large abortion rates (unpublished observation).

### Treatments

We made three treatments. In the 2Cf treatment, individual figs were protected in fine mesh bags early in development to preclude parasite oviposition. When the figs became receptive, 2 pollinators were introduced into each fig. When the figs are receptive, they attract pollinators that fly around them. In this experiment, we caught the pollinators in the air with a large fine mesh bag. We then used a fine paintbrush to pick the pollinator and deposit it on the surface of the fig. The wasps rapidly find their way to the ostiole. To ensure wasp vitality, all pollinator introductions were performed in the morning. After the pollinator introduction, the figs were bagged again. When the wasp offspring emerged from the figs, wasps and figs were collected in 70% alcohol. The figs were then opened and numbers of developed galls, seeds, and insects were counted.

In the St treatment, the figs were exposed to oviposition by *S. testacea* but not to pollinator oviposition. In this treatment, the figs were only bagged after the period of oviposition by *S. testacea* so that no other fig wasp species oviposited in them. Large numbers of figs aborted in this treatment, and we checked and cleaned the dropped figs every 2 or 3 days. Male *S. testacea* does not cut exit holes from the fig. So, in this treatment, when the wasps were ready to emerge from their galls, we cut a small hole near the ostiole and let the wasp emerge. Wasps and figs were then collected in 70% alcohol. Then the figs were opened and numbers of developed galls and insects were counted.

In the St + 2Cf treatment, the figs were first exposed to oviposition by *S. testacea* and then they were protected in bags. At fig receptivity, two pollinators were introduced per fig as in the first treatment. When the wasps emerged from the figs, wasps and figs were collected in 70% alcohol. The figs were then opened and numbers of developed galls, seeds, and insects were counted.

The length (axis length, distance from the pedicel to the bracts of the fig) and width (the longest diameter and shortest diameter of the fig) of mature figs were measured for the three treatments to estimate fig size. The following development ratio will designate the ratio between the number of wasps and the number of galls. The assumption is that empty galls correspond to galls that contained a wasp larva that died during development.

### Data analysis

We use SPSS 17.0 to do all the statistics. To compare the difference of fig volume and development ratio among treatments, one-way ANOVA was used in the analysis. The experiment was carried out on two trees, so we use a GLM model to test the tree effect. No significant effect of the tree was found, so we don’t display them in the results. In order to test the effect of *S. testacea* on the wasp production of pollinators and seed production, an independent T-test was used to compare the difference of gall number, wasp offspring number and seed number between the two treatments that included pollinators.

We use the development ratio of *S. testacea* in St treatment and development ratio of *C. fusciceps* in Cf treatment to predict the theoretical total galls for the 2Cf + St treatment (predicted gall number = *C. fusciceps* offspring number in 2Cf + St treatment/development ratio of *C. fusciceps* in Cf treatment + *S. testacea* offspring number in 2Cf + St treatment/development ratio of *S. testacea* in St treatment), and use independent T-test to compare the difference between predicted and actual values of gall number, so as to determine the actual reason why the development ratio declines with the involvement of *S. testacea* in 2Cf + St treatment compared with 2Cf treatment*.*

## Data Availability

Data are available from the authors on request.
